# Primary Angiosarcoma of Bone of the Skull Base: A Case Report and Literature Review

**DOI:** 10.7759/cureus.98464

**Published:** 2025-12-04

**Authors:** Muhammad Rashid Hanif, Saif ur Rab, Nausheen Yaqoob, Sharjeel Usmani, Boris Itkin

**Affiliations:** 1 Medical Oncology, Sultan Qaboos Comprehensive Cancer Care and Research Centre, Muscat, OMN; 2 Pathology, Sultan Qaboos Comprehensive Cancer Care and Research Centre, Muscat, OMN; 3 Nuclear Medicine, Sultan Qaboos Comprehensive Cancer Care and Research Centre, Muscat, OMN

**Keywords:** aim chemotherapy, epithelioid angiosarcoma, fdg-pet, pab, paclitaxel

## Abstract

Primary angiosarcoma of bone (PAB) is an exceedingly rare entity characterized by aggressive clinical behavior. Following surgical removal, systemic relapse is common, and the overall prognosis is poor. Several systemic therapy agents and combinations have been used in the treatment of PAB. However, the evidence for their efficacy is limited to retrospective case series and case reports, making it difficult to assess their relative effectiveness. We report a case of systemically relapsed PAB along with a literature review aimed at increasing the available information on PAB responses to different types of systemic therapy.

## Introduction

Primary angiosarcoma of bone (PAB) is a high-grade malignant tumor with endothelial differentiation [1]. Approximately 4% of all angiosarcomas arise in the bone, accounting for less than 1% of the malignant tumors of the bone. It can occur at any age with a male predominance [2]. PAB appears most often in adults from the second to the seventh decade of life, and it can affect any portion of the skeleton, although the long bones of the extremities and the axial skeleton are mostly involved [3]. It may be multifocal at presentation and often involves the adjacent skeletal sites. Most of the lesions arise in the bone; however, they may occur after radiation or be associated with bone infarction as well [4]. This tumor exhibits aggressive characteristics. Diagnosing high chances of local recurrences and distant metastasis and treating them is difficult and necessitates a multimodal approach that includes chemotherapy, radiation therapy, and surgical resection.

## Case presentation

A 39-year-old woman presented with headache and weight loss for several months. MRI of the brain revealed a 3.6 cm mass in the spheno-occipital region extending to the nasal cavity, nasopharynx, and clivus, encasing the pituitary. She underwent transsphenoidal resection of the tumor.

**Figure 1:**
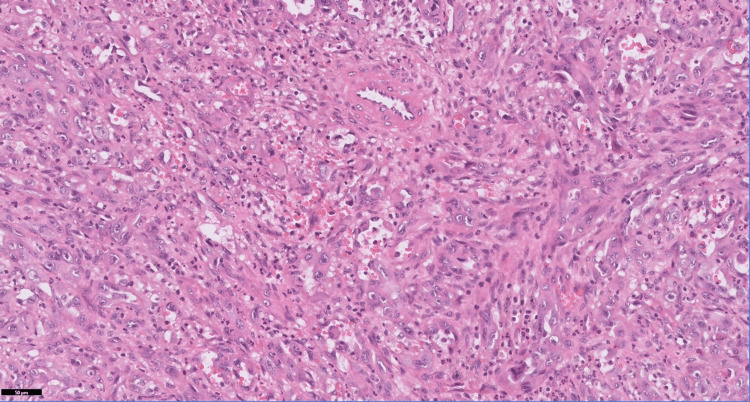
High-power magnification showing anastomosing tumor channels with hyperchromatic, pleomorphic nuclei. Note the tripolar mitotic figure (H&E 40X).

Histopathological examination revealed a high-grade malignant neoplasm organized in nests and fascicles, infiltrating between bone trabeculae. Figure 1 shows high-power magnification with anastomosing tumor channels and hyperchromatic, pleomorphic nuclei, including a tripolar mitotic figure (H&E, 40X).

**Figure 2:**
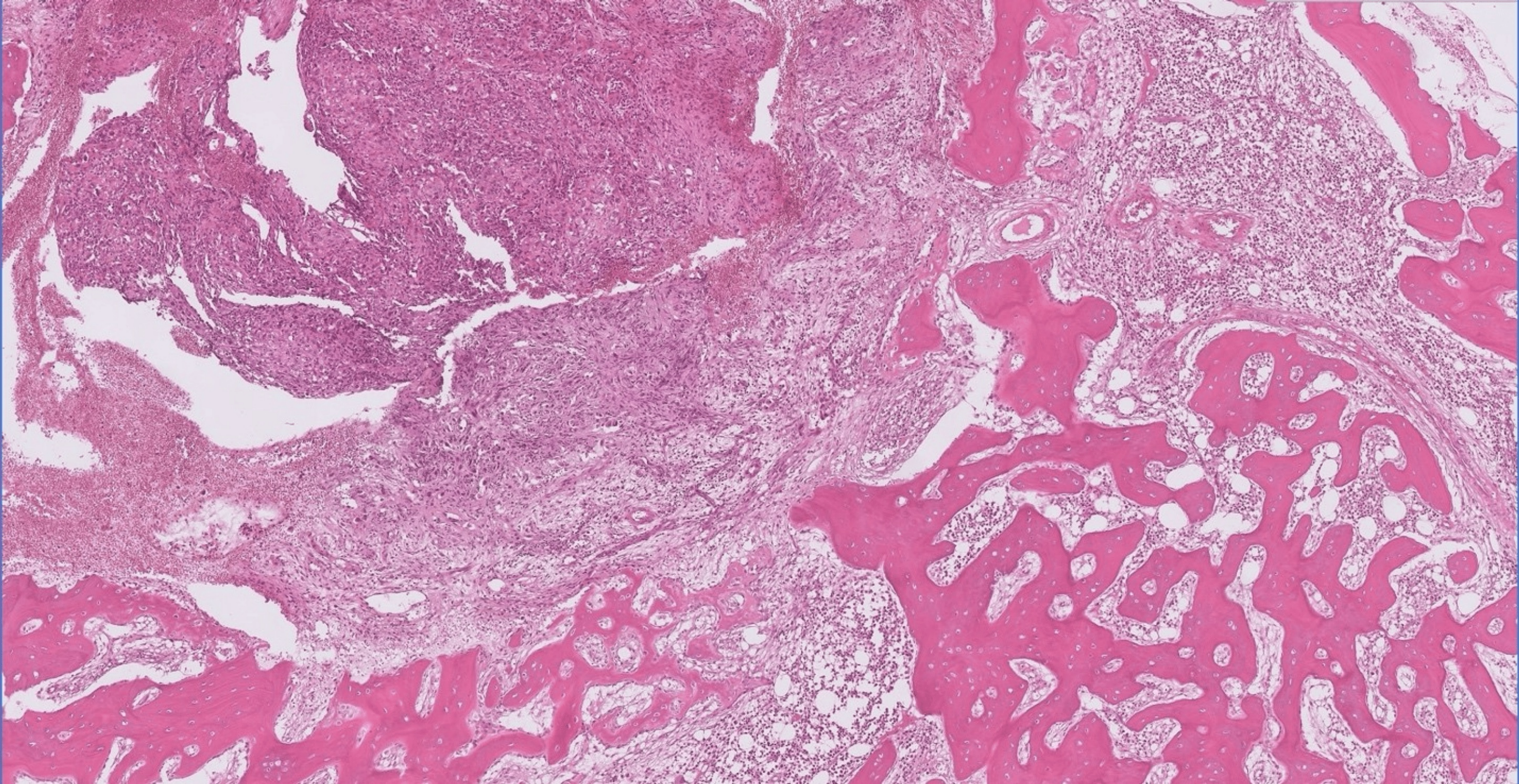
Low-power magnification showing a tumor infiltrating between bone trabeculae (H&E 20X).

At low power, the tumor was seen infiltrating between bone trabeculae, as shown in Figure 2 (H&E, 20X). 

**Figure 3:**
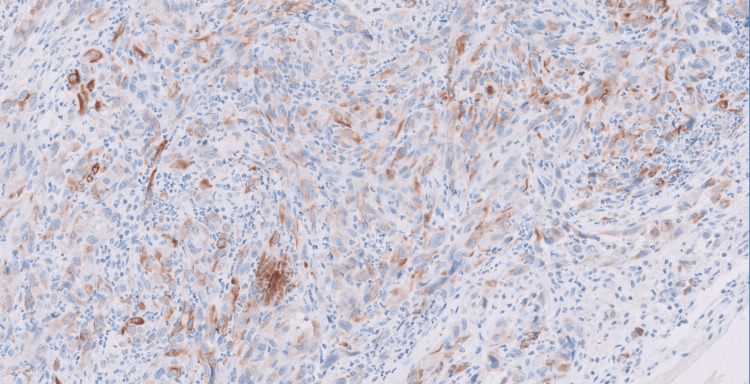
Tumor cells showing patchy positivity for CKAE1/AE3 antibody.

Tumor cells demonstrated patchy positivity for CKAE1/AE3 antibody, as shown in Figure 3 (immunohistochemistry, 40X). 

**Figure 4:**
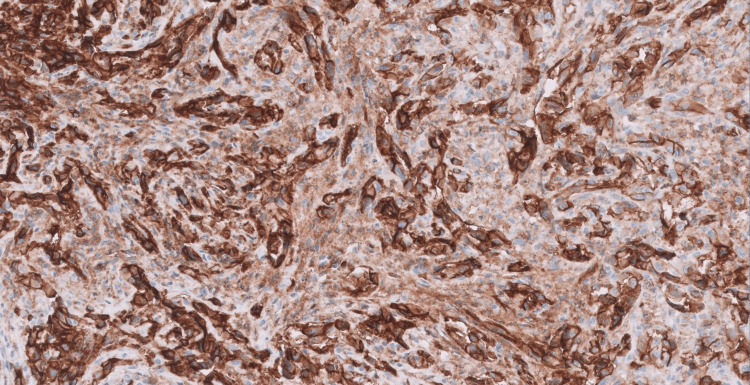
Tumor cells staining positive for the immunohistochemical stain CD31.

Tumor cells were also diffusely and strongly positive for CD31, as shown in Figure 4 (immunohistochemistry, 40X). 

**Figure 5:**
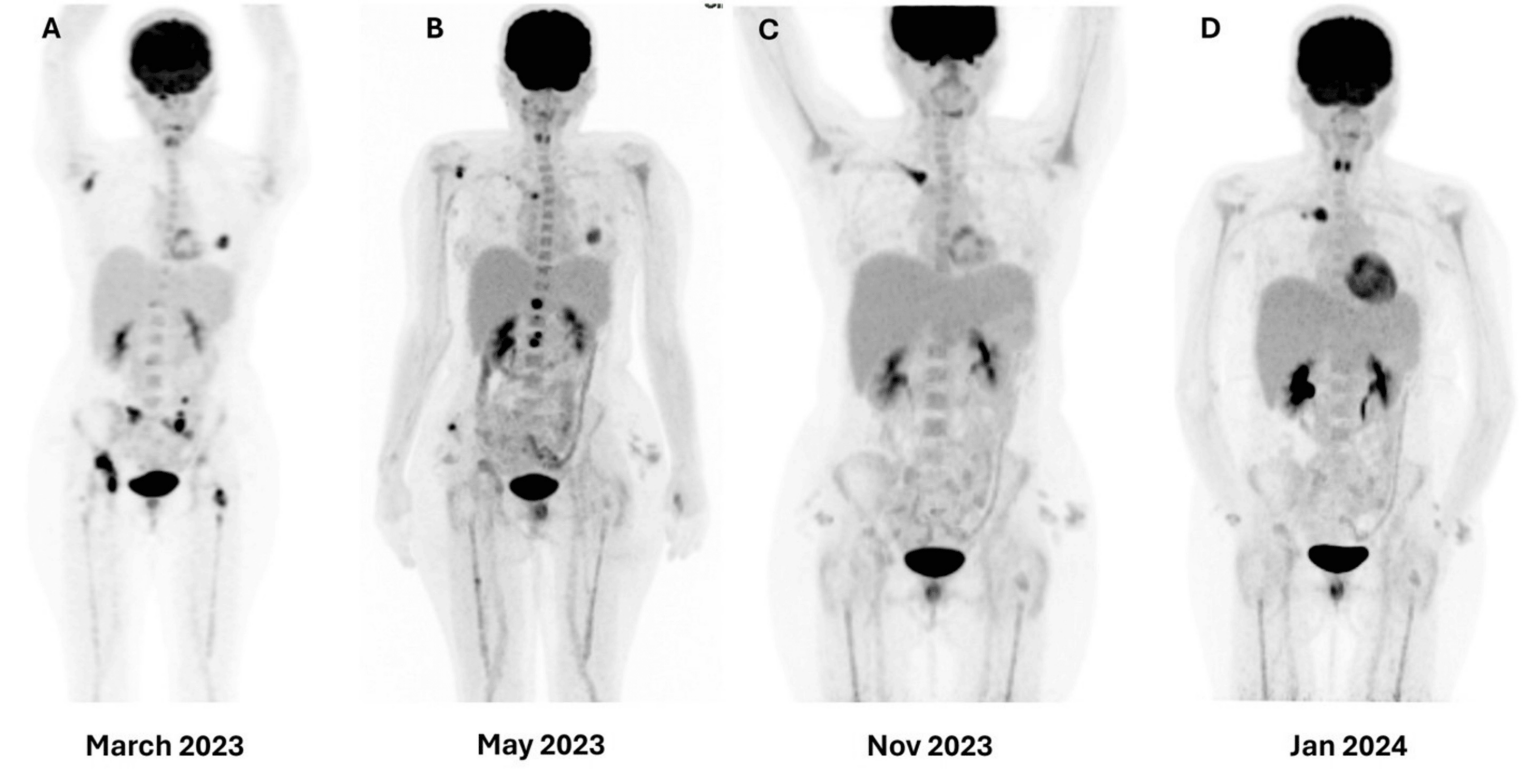
FDG-PET-CT scans at different intervals during the treatment.

Three months after surgery, the headache recurred, accompanied by pain in the hip bones. The FDG-PET scan (08.03.2023) showed multiple hypermetabolic, predominantly lytic bone lesions in the right occipital condyle, right scapula, left humeral head, left fourth rib, several vertebrae, pelvis, and hips bilaterally. Additionally, non-specific increased uptake was noted in the nasopharynx. Figure 5 illustrates FDG-PET-CT scans at different intervals during treatment. 

**Figure 6:**
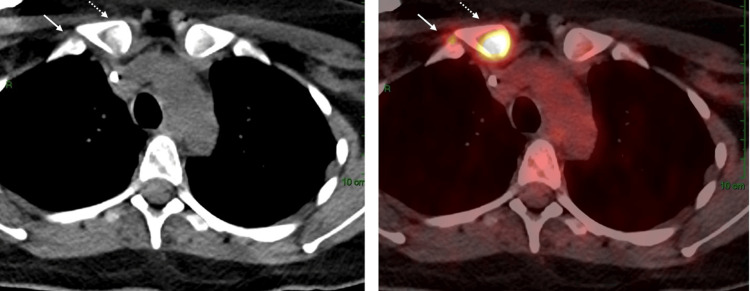
A PET-CT scan shows a new focus of uptake in the right first rib contiguous to the right clavicle.

A subsequent PET-CT scan showed a new focus of uptake in the right first rib contiguous to the right clavicle, as shown in Figure 6. 

She received palliative radiotherapy (20 Gy in 5 fractions to the skull base and hips, plus 8 Gy single fraction to the rib). Weekly paclitaxel was initiated abroad, but after two months, the PET scan revealed progression. Second-line AIM chemotherapy (doxorubicin + ifosfamide + mesna) induced a partial response after three cycles, consistent with prior reports of anthracycline efficacy. After six cycles, oligo-progression occurred in the clavicle, treated with radiotherapy (8 Gy in a single fraction).

Later, pazopanib was administered for four months, providing modest symptom control, consistent with exploratory evidence of antiangiogenic therapy in angiosarcoma. The disease progressed rapidly thereafter, and the patient passed away.

## Discussion

Approximately 4% of all angiosarcomas arise in the bone, accounting for less than 1% of the malignant tumors of the bone [5,6]. These tumors may be multifocal at presentation and often involve the adjacent skeletal sites. Most of the lesions arise in the bone; however, they may occur after radiation or be associated with bone infarction. This tumor has an aggressive nature and carries a high risk of recurrence and distant metastasis [7]. The diagnosis and treatment may be challenging and require a multimodality approach, which comprises chemotherapy, radiation therapy, and surgical resection.

Histopathological examination of angiosarcomas ranges from well-formed, anastomosing vascular channels to solid sheets of high-grade epithelioid or spindled cells without clear vasoformation. It varies in its degree of cytological atypia and architectural differentiation. Vasoformative areas are composed of ramifying channels lined by spindle or epithelioid cells with intraluminal budding, or papillary-like projections. Tumor cells have abundant eosinophilic to amphophilic cytoplasm, large vesicular nuclei, and prominent nucleoli. Nuclear atypia, brisk mitotic activity, and coagulative necrosis are common. Sometimes tumors have low-grade morphology, with well-formed vascular channels lined by minimally atypical spindled cells. Epithelioid angiosarcoma is a rare histopathologic variant of angiosarcoma characterized by an epithelioid morphology. This subset can histologically mimic non-vascular neoplasms and pose serious challenges in diagnosing them correctly. Epithelioid angiosarcomas typically have a solid architecture with only focal vasoformative channels, showing diffuse, sheet-like patterns of large, atypical epithelioid or polygonal cells with ovoid vesicular nuclei, prominent large central nucleoli, and abundant cytoplasm, as seen in our case. On immunohistochemical staining, they show membranous CD31 and nuclear ERG positivity, with variable expression of CD34 [2,4].

Due to the heterogeneity and rarity of this tumor, high-quality evidence is lacking for management. For localized and resectable cases, complete resection, if possible, is the treatment of choice, with a possible small DFS benefit with adjuvant radiotherapy [5]. In metastatic cases, although systemic chemotherapy can induce modest responses, the overall prognosis remains very poor, with a five-year survival rate of only 8% as reported by Palmerini et al. in 2014. The authors concluded that anthracycline and paclitaxel may have beneficial effects on PAB, but prospective validation is needed.

In the largest retrospective study on PAB by Palmerini et al. in 2020, a significant proportion of patients (44%) presented with metastatic disease [8]. Out of 18 patients, eight patients (44%) received doxorubicin-ifosfamide, and four patients (22%) received an osteosarcoma-like regimen (doxorubicin, methotrexate, cisplatin, ifosfamide) as first-line treatment. The response rates for these regimens were similar. Other cytotoxic agents used in the remaining patients included paclitaxel (n=3, 17%), gemcitabine (n=2, 11%), and pegylated liposomal doxorubicin (n=1, 6%). The impact of treatment response on one-year survival was significant. The one-year overall survival rate was 67% for those with a partial response or stable disease (n=7) and 18% for those with progressive disease (n=11), with a p-value of 0.002. We reported a case of PAB that was refractory to the first-line paclitaxel but responsive to the second-line AIM chemotherapy. In our case, the patient maintained a good treatment response (partial response after three cycles) and then developed oligo-progression.

Beyond cytotoxic chemotherapy, targeted therapy with antiangiogenic agents and multi-kinase inhibitors (e.g., bevacizumab, pazopanib, sorafenib) has demonstrated modest activity in soft tissue angiosarcomas, with response rates of approximately 15%. Weekly paclitaxel has also shown efficacy in unresectable angiosarcoma, as demonstrated in the ANGIOTAX study, where durable responses were achieved in a subset of patients [9]. While these data are derived primarily from soft tissue angiosarcomas, they provide exploratory evidence that such agents may have a role in PAB, particularly in later treatment lines when conventional chemotherapy options are exhausted. However, as highlighted by Young et al., angiosarcoma remains a highly aggressive malignancy with poor outcomes despite multimodal therapy, underscoring the urgent need for prospective trials and novel therapeutic strategies [10].

## Conclusions

PAB is an ultra-rare and aggressive tumor with a generally poor prognosis. Due to the heterogeneous nature of this rare tumor entity and the lack of high-quality evidence, both diagnosis and treatment can be challenging. Various chemotherapeutic agents may induce a response, although data are insufficient to recommend the superiority of any regimen. Palliative radiotherapy should be considered for symptomatic sites. Prospective trials are necessary to identify more effective and definitive treatment options.
